# Active Peptides Derived from Snail Mucus Promoted Wound Healing by Enhancing Endothelial Cell Proliferation and Angiogenesis

**DOI:** 10.3390/ijms262110341

**Published:** 2025-10-23

**Authors:** Guanqiang Li, Yucheng Shi, Junmei Zhu, Kehan Zhu, Bo Hu, Xianchen Huang, Yuan Sun, Duxin Li, Xicheng Zhang

**Affiliations:** 1 Department of Vascular Surgery, The Medical Center of Soochow University, Suzhou 215000, China13405093661@163.com (J.Z.);; 2 College of Pharmaceutical Sciences, Soochow University, Suzhou 215021, China

**Keywords:** snail mucus, active peptides, endothelial cells, proliferation wound healing

## Abstract

Snail mucus has shown promise in promoting wound healing; however, its active components and their mechanisms of action are poorly understood. In the present study snail mucus was isolated and hydrolyzed using trypsin to obtain snail mucus active peptides (SMAPs). SMAPs were analyzed using liquid chromatography–mass spectrometry, and bioinformatics screening. Among the screened peptides, an active 12-amino-acid peptide, EK-12 (molecular weight: 1366.2 Da), was identified and synthesized using a solid-phase peptide synthesis approach. In vitro functional verification showed that EK-12 significantly promoted endothelial cell proliferation, migration, and tube formation. An in vivo experiment demonstrated that EK-12 significantly accelerated wound healing in mouse models. Pathological examination revealed a significantly upregulated expression of CD31 and vascular endothelial growth factors in wound tissues, suggesting that this is the mechanism by which the active peptide promotes angiogenesis and wound healing. Thus, snail mucus-derived peptides hold strong potential for development as therapeutic agents for wound healing.

## 1. Introduction

The skin is the largest organ in the human body and serves as a vital barrier against external injuries and infections [1]. Damage to this organ is usually caused by trauma, surgery, infection, or diseases such as diabetes mellitus. However, the natural healing process of skin injury is relatively slow, and wounds are often prone to infection. Thus, the identification of bioactive components and elucidation of their therapeutic mechanisms are major focuses of current biomedical research [2].

Snail mucus has a long history of application in wound management in humans. Our previous review of its chemical composition found that snail mucus contains abundant natural collagen, allantoin, proteins, and glycosaminoglycans [3]. Recent studies have revealed that mucin and polysaccharides promote wound healing by functioning as adhesive agents [4,5]. Furthermore, snails—shaped by diverse ecological niches and evolutionary pressures—have emerged as promising sources of antimicrobial peptides and proteins (AMPs) [6]. However, skin injury repair and regeneration are a complex process consisting of four stages: hemostasis, inflammation, proliferation, and remodeling [7]. The process entails the activation of various cells, such as nerve cells, endothelial cells, mast cells, macrophages, neutrophils, keratinocytes and fibroblasts, which all come into play through a plethora of cytokines and growth factors [8]. Angiogenesis is crucial for wound healing, as local injury-induced hypoxia and inflammatory factors stimulate EC proliferation and migration, gradually forming new epithelium, blood vessels, and granulation tissue. Orchestrated by a precise coordination of pro-angiogenic factors such as VEGF, the newly formed vasculature not only supplies oxygen, nutrients, and immune mediators to the hypoxic wound microenvironment but also facilitates the clearance of catabolites. This functional angiogenesis is a fundamental pillar of successful wound healing [9,10,11,12].

Current evidence indicates that snail mucus enhances fibroblast proliferation and migration, potentially through the induction of interleukin-8 (IL-8) and other unidentified growth factors, thus facilitating wound closure, either directly or indirectly [13]. Furthermore, previous studies have shown that snail mucus-treated wounds demonstrate upregulated expression of key angiogenic markers, such as CD31 and vascular endothelial growth factor (VEGF), along with enhanced matrix deposition, indicating its regulatory role in angiogenesis [14]. Despite these advances, the specific bioactive components and molecular mechanisms underlying the therapeutic effects of snail mucus remain poorly understood. Therefore, the present study aimed to screen snail mucus active peptides (SMAPs) for wound healing and explore their potential mechanisms. These results provide a basis for the development of natural drugs for wound healing.

## 2. Results

### 2.1. Screening and Synthesis of SMAPs

**Figure 1:**
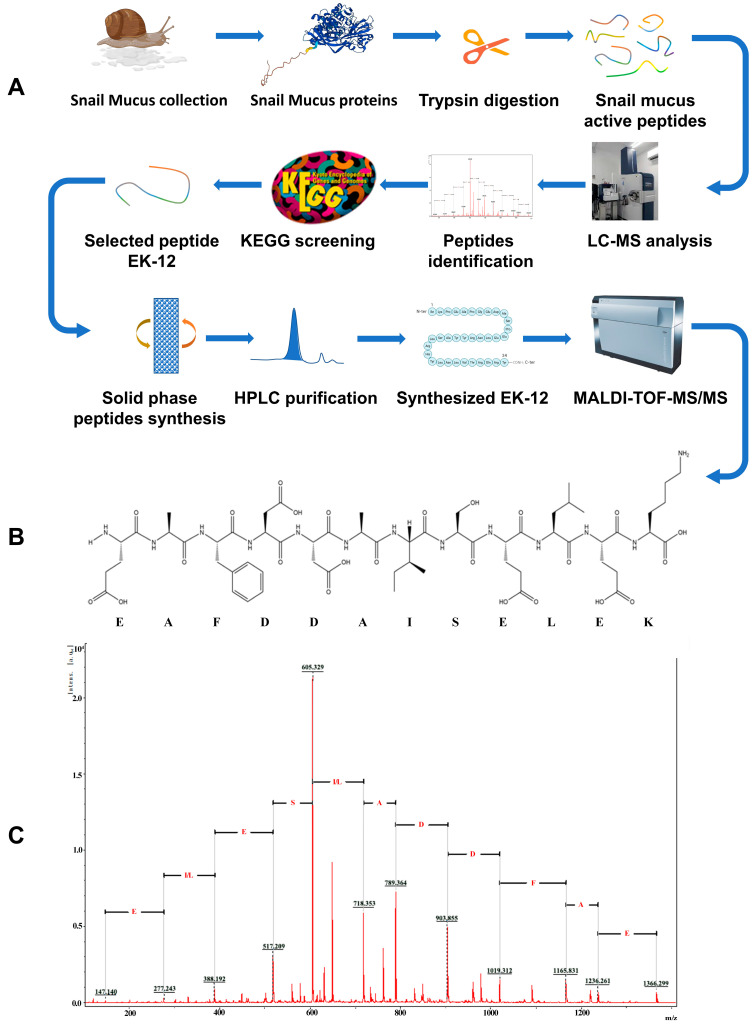
Screening and synthesis procedures of the SMAP. (**A**) Procedure for screening and synthesis of SMAP; (**B**) Synthetic EK-12 amino acid sequence; (**C**) MS/MS spectrum of the synthesized EK-12.

**Figure 2:**
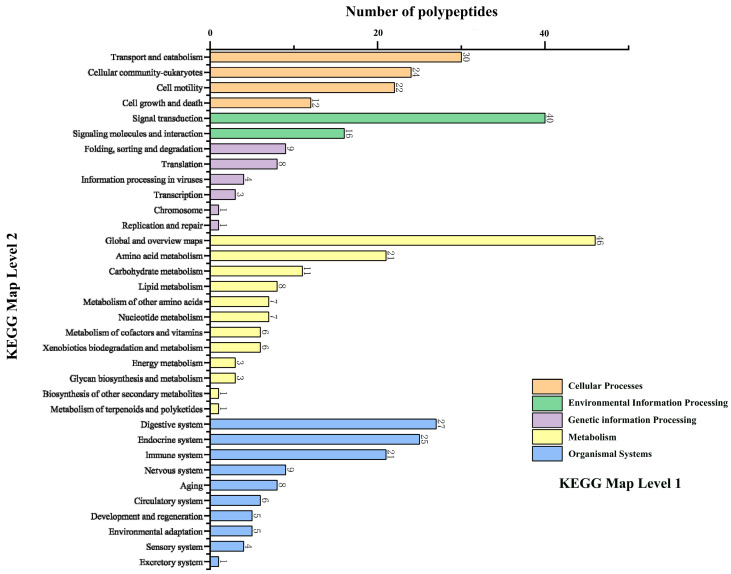
Results of KEGG pathway enrichment analysis.

Proteins were isolated from snail mucus and subjected to trypsin digestion to generate the SMAPs. Through a standardized proteomics process (Figure 1A), 621 candidate peptides were identified from the SMAPs and functionally annotated. Eighty-eight peptides were found to be involved in basic Cellular Processes, and 12 peptides were further identified as directly participating in the regulation of the “Cell Growth and Death” pathways (Figure 2). Because small molecule peptides are readily transported across the membrane and have relatively high bioavailability, a molecular weight of <3000 Da was set as the screening threshold. Among the 12 candidate peptides, EK-12 (N-terminal sequence: EAFDDAISELEK, Figure 1B) with a molecular weight of 1366.2 Da was chosen. It was synthesized using the solid-phase peptide synthesis method and purified by HPLC (C18 column, acetonitrile/water gradient elution) to a purity of ≥98%. The sequence of the synthesized EK-12 was confirmed by tandem mass spectrometry (MS/MS) (Figure 1C).

### 2.2. Effects of EK-12 on Cell Proliferation

**Figure 3:**
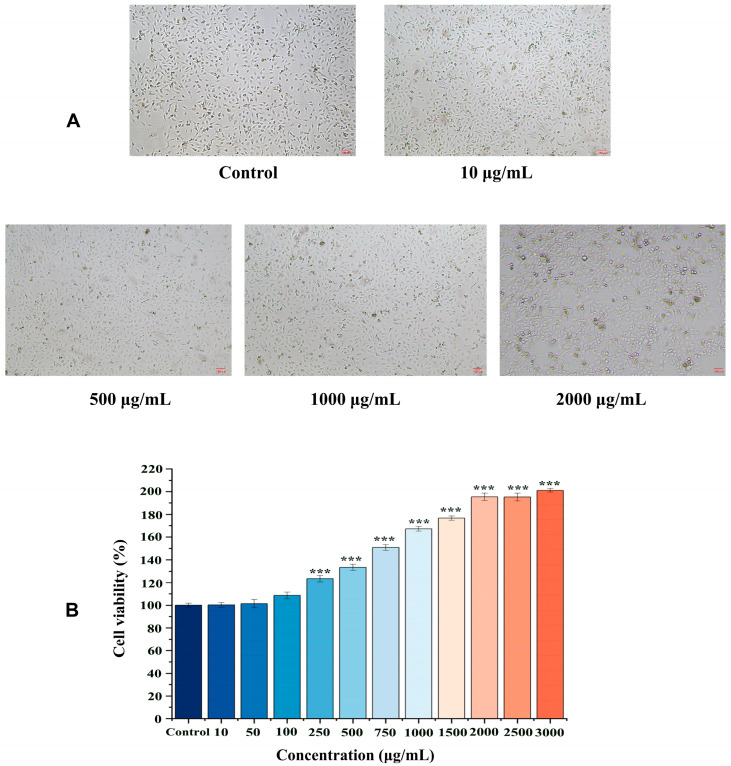
EK-12 promoted HUVEC proliferation in a concentration-dependent manner. (**A**) Phase-contrast images illustrating the morphology of the HUVECs (40×). (**B**) Quantitative analysis of cell proliferative activity across all tested concentrations compared to the control group (*n* = 3 experimental replicates; *** *p* < 0.001 vs. Control).

Compared to the control group, the CCK-8 assay (absorbance at 450 nm) demonstrated a significant increase in the proliferative activity of HUVECs treated with each SMAP in the experimental group (*p* < 0.01). This proliferative effect was concentration-dependent, showing a significant increase with increasing concentrations of EK-12 (*p* < 0.01). Notably, at 1500 μg/mL EK-12, the proliferative activity reached a plateau, as no statistically significant difference was observed between the 1500 and 3000 μg/mL concentrations (*p* > 0.05), indicating a saturation effect at 1500 μg/mL (Figure 3, Supplementary Materials).

### 2.3. Effects of EK-12 on Cell Migration

**Figure 4:**
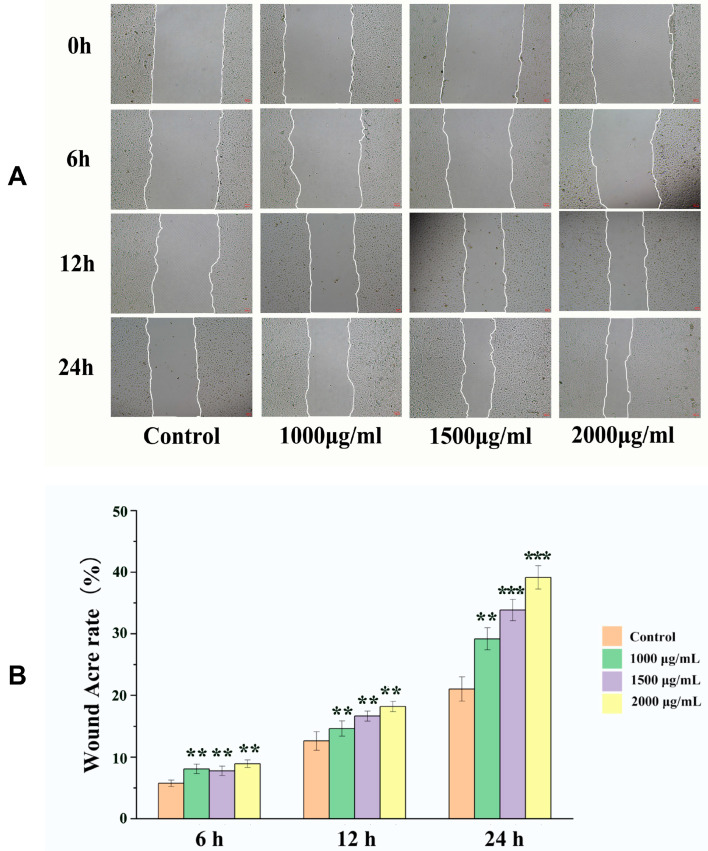
EK-12 promoted HUVECs migration. (**A**) Representative phase-contrast images of scratch wounds at 0–24 h post-treatment (40×); (**B**) Treatment with EK12 at 1000, 1500, and 2000 μg/mL significantly increased HUVECs migration rates compared to the control group (*n* = 3 experimental replicates; ** *p* < 0.01, *** *p* < 0.001 vs. Control).

Based on the CCK-8 results, EK-12 concentrations of 1000, 1500, and 2000 μg/mL were selected for the cell scratch assays. All concentrations significantly increased HUVECs migration rates compared to the blank control group at 6, 12, and 24 h post-scratch (*p* < 0.001), with concentrations > 1500 μg/mL exerting a more significant effect; however, the 1500 and 2000 μg/mL groups showed no significant differences (*p* > 0.05) (Figure 4).

### 2.4. Effects of EK-12 on Tube Formation Capacity

**Figure 5:**
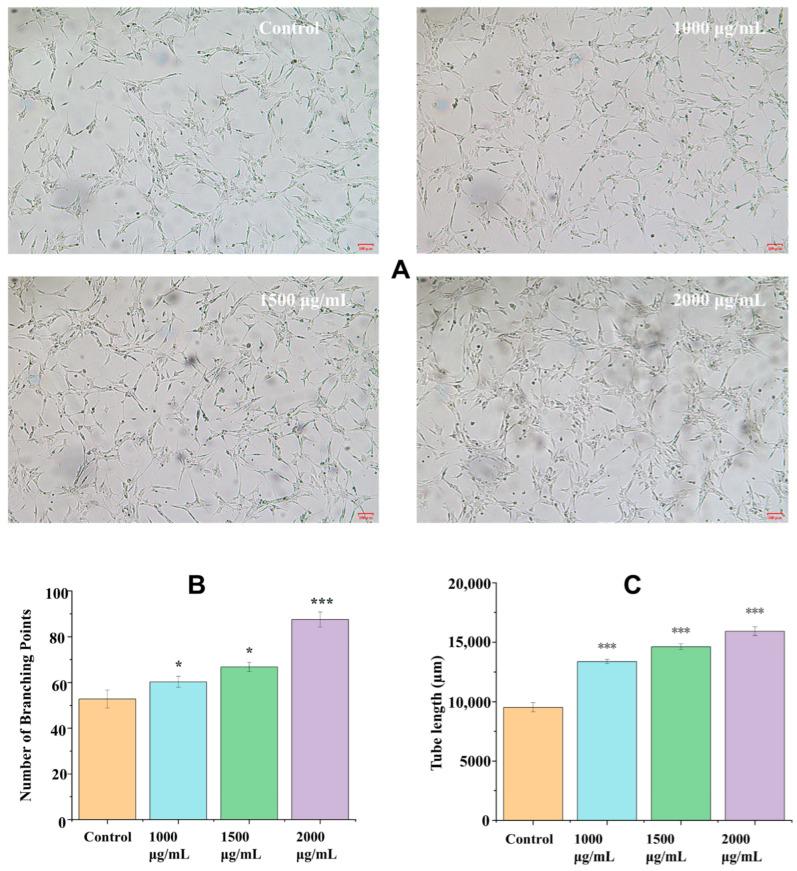
EK-12 promotes tube formation capacity (24 h treatment). (**A**) Microscopic images of tubular network formation by HUVECs treated with varying EK-12 concentrations (40×). (**B**) Tube formation ability (number of branching points) at different concentrations. (**C**) Quantitative analysis of average tube length. (*n* = 3 experimental replicates; * *p* < 0.05, *** *p* < 0.001 vs. Control).

Compared with the observation in the saline control group, EK-12 at 1000, 1500, and 2000 μg/mL significantly increased the number of ECs in the experimental group, forming tubular structures on Matrigel after 24 h in a concentration-dependent manner. However, no statistically significant difference was observed between the 1500 μg/mL and 2000 μg/mL groups (*p* > 0.05; Figure 5).

### 2.5. Effects of EK-12 on Wound Healing and Angiogenesis

**Figure 6:**
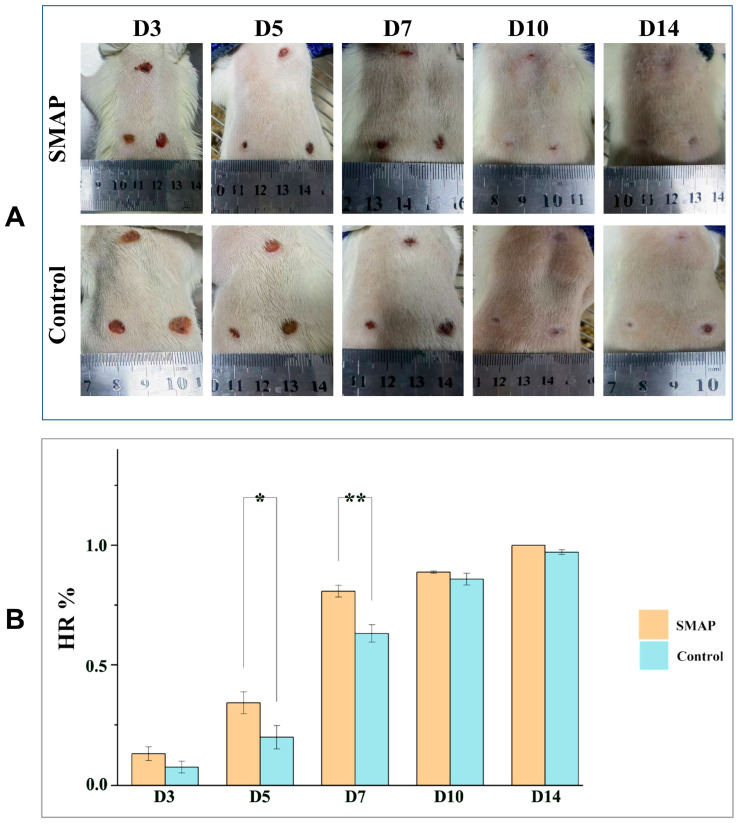
SMAPs accelerated wound healing in a mouse model. (**A**) Macroscopic images of wound healing progression in the EK12-treated and control groups. (**B**) On days 5 and 7, The HR in the EK-12 group was significantly higher than that in the control group (*n* = 2 experimental replicates; * *p* < 0.05, ** *p* < 0.01 vs. Control).

**Figure 7:**
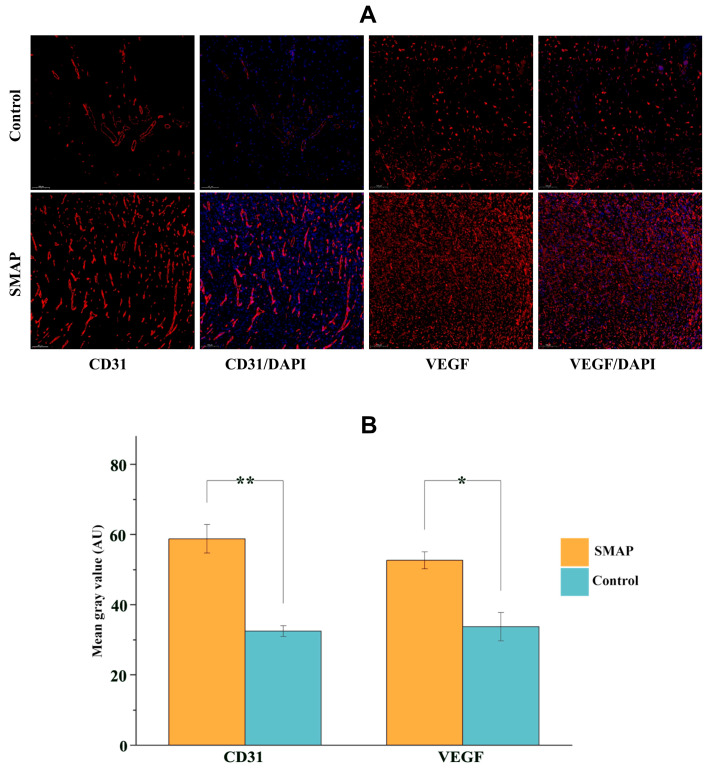
Pathological staining of the wound tissue revealed that SMAPs upregulated angiogenic markers. (**A**) Immunofluorescence staining of CD31, VEGF, and DAPl in SMAP-treated and control tissues stained red and blue colors, respectively (15×). The SMAP-treated samples exhibited markedly stronger fluorescence signals for both markers. (**B**) Semi-quantitative analysis of CD31 and VEGF expression (mean gray values) confirmed significant upregulation in the APs group (* *p* < 0.05, ** *p* < 0.01 vs. Control).

The wounds in the EK-12 group healed completely within 14 days, and the healing speed was significantly faster than that in the Vaseline control group (Figure 6A,B). On days 5 and 7, the HR in the EK-12 groups was significantly higher than that in the control group (*p* < 0.05, *p* < 0.01 for days 5 and 7, respectively). On days 10 and 14, most wounds were nearly healed, and no significant difference was observed in HR between the two groups (*p* > 0.05). Immunofluorescence analysis further revealed elevated expression of CD31 (endothelial marker) and VEGF (angiogenic factor) in SMAP-treated tissues, as evidenced by higher mean fluorescence intensities (*p* < 0.05 and *p* < 0.01, respectively; Figure 7).

### 2.6. Expression of VEGFA and CD31 in Wound Tissues

**Figure 8:**
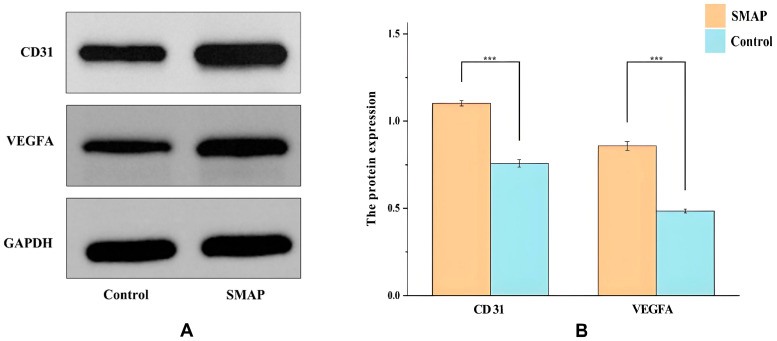
SMAP upregulates VEGFA and CD31 in wound tissues. (**A**) Representative Western blot images of VEGFA, CD31, and GAPDH (loading control) in the Control and SMAP-treated groups. (**B**) Quantitative analysis of protein expression by normalizating to GAPDH (*n* = 5; data are presented as means ± SD; *** *p* < 0.001 vs. Control).

Western blot analysis showed that SMAP treatment significantly increased the expression of angiogenesis-related proteins in wound tissue (Figure 8). After normalization against GAPDH, VEGFA expression in the SMAP group was significantly higher than that in the control group (*p* < 0.01), and CD31 expression was also increased (*p* < 0.05) (Figure 8B). VEGFA and CD31 signal intensities were higher in the SMAP group than in the control group (Figure 8A). The expression of GAPDH (the internal reference) did not differ between the two groups, supporting the reliability of our results. These results indicate that SMAP promotes wound angiogenesis by upregulating VEGFA/CD31.

## 3. Discussion

In chronic wounds, such as diabetic foot ulcers, persistent inflammation, microcirculatory dysfunction, and impaired EC activity collectively suppress angiogenesis and extracellular matrix remodeling, significantly hindering healing [14]. Thus, protecting ECs from apoptosis and promoting localized vascularization are pivotal strategies for wound management [15]. Therefore, this study used ECs to evaluate the effects of SMAP on wound healing.

Snail mucus contains abundant bioactive peptides that exhibit both cell proliferation–promoting and antibacterial effects [11,16]. In the present study, an active peptide derived from snail mucus was screened and synthesized for the first time, and it significantly enhanced the proliferation, migration, and tube formation of ECs. In vivo, EK-12 treatment upregulated VEGF and CD31 expression, facilitating collagen deposition and capillary network formation, which improved local blood perfusion and accelerated wound healing [17].

Mechanistically, the significant upregulation of VEGFA (a key angiogenic factor) and CD31 in EK-12-treated wound tissues (Figure 7 and Figure 8), coupled with enhanced proliferation, migration, and tube formation of ECs in vitro (Figure 2, Figure 3 and Figure 4), strongly suggests that EK-12 promotes angiogenesis by modulating the VEGF signaling pathway. Given that VEGF exerts its effects primarily via VEGFR-2 binding [18,19,20], we hypothesized that EK-12 may interact with VEGFR-2 or its downstream effectors and trigger downstream signaling pathways, such as PI3K/AKT and MAPK/ERK. These pathways regulate cell cycle progression, survival, and angiogenesis, ultimately contributing to the formation of a functional vascular network [18,19,20]. However, direct evidence of receptor binding (e.g., via docking studies or biochemical assays) is currently lacking and will be explored in future studies.

These findings highlight the potential of SMAP as therapeutic agents for ischemic ulcers. Furthermore, this study demonstrated that EK-12 promotes cell proliferation in a concentration-dependent manner. Cellular proliferative and migratory activities increased with increasing EK-12 concentrations; however, beyond a certain saturation, concentration and cellular activity plateaued. This suggests that EK-12, at higher concentrations, may either activate a negative feedback mechanism that regulates cell proliferation or achieve receptor binding saturation, thereby preventing further enhancement of proliferative activity despite additional increases in concentration.

Furthermore, although this study focused on angiogenesis, snail mucus contains anti-inflammatory components [9,13]. Separately, anti-inflammatory effects have been widely recognized as playing an essential role in the wound healing process [21]. Chronic inflammation impedes wound healing, and accelerated vascularization induced by EK-12 may indirectly mitigate inflammation by improving tissue oxygenation and metabolic waste removal [22]. Whether EK-12 directly modulates inflammatory cytokines (e.g., IL-1β, TNF-α, or IL-10) requires further investigation.

It is believed that the snail mucus also contains various unidentified active peptide components and synergistic factors, which collaborate to promote wound healing [13]. Future studies should focus on isolating and synthesizing additional peptides, characterizing their biological functions, and elucidating their molecular mechanisms. These efforts may provide new insights into the treatment of ischemic vascular disease and regenerative medicine.

Nevertheless, this study had some limitations. First, the research primarily utilized a healthy mouse wound model, which may not fully replicate the complex pathological microenvironment of chronic human wounds, such as diabetic ulcers, which are characterized by persistent inflammation and impaired angiogenesis. Second, although the upregulation of CD31 and VEGF suggests a pro-angiogenic mechanism, the specific intracellular signaling pathways activated by EK-12 (e.g., PI3K/AKT or MAPK/ERK) have not been experimentally validated. Moreover, while immunofluorescence and IHC provided robust spatial quantification of VEGF and CD31 expression, future studies employing Western blotting will further validate total protein levels in homogenized wound tissues. Finally, the long-term biosafety profile and potential immunogenicity of the synthetic EK-12 peptide require further evaluation in extended studies before it can be translated into clinical applications.

## 4. Materials and Methods

### 4.1. Preparation of Snail Mucus Lyophilized Powder

Fresh adult *Achatina fulica* snails weighing over 20 g were provided by Qianfu Company (Jiaxing, China). They were repeatedly rinsed with tap and distilled water and placed in a rotating drum to stimulate mucus secretion. The collected mucus was dissolved in ultrapure water, centrifuged at 5000× *g* for 10 min, and filtered through a 0.45-μm aqueous membrane to remove any impurities. The supernatant was centrifuged at 3000× *g* for 10 min, lyophilized, and stored as lyophilized powder.

### 4.2. Enzymatic Hydrolysis of Snail Mucus

Lyophilized snail mucus powder (300 mg) was dissolved in 30 mL of 0.2 M disodium hydrogen phosphate-0.1 M citrate buffer (pH 6.8). Trypsin (6 mg, 2500 U/mg activity) was added, and the mixture was incubated in a water bath at 37 °C for 4 h. Subsequently, 10 mL of the reaction solution was heated at 100 °C for enzyme inactivation and centrifuged at 5000× *g* for 10 min to remove the enzymes. The supernatant was collected and lyophilized to obtain a mixture of SMAPs.

### 4.3. Screening and Synthesis of SMAPs

The protein mixture of enzymatically hydrolyzed snail mucus was subjected to a standardized liquid chromatography–mass spectrometry (Bruker tims TOF, Saarbrucken, Germany) for proteomics studies and bioinformatics analysis. Kyoto Encyclopedia of Genes and Genomes (KEGG) pathway enrichment analysis was performed to annotate the functions of the identified peptides. Peptides involved in the “Cell Growth and Death” regulatory pathway were selected based on their molecular weight and synthesized by Temic Biotech (Suzhou, China). The peptides were purified using high-performance liquid chromatography (HPLC) and verified by mass spectrometry for molecular weight consistency (molecular weight error < 0.1 Da). The peptides were stored as lyophilized powder at −20 °C (Figure 1A).

### 4.4. Cell Culture and Proliferation Assay

Human umbilical vein endothelial cells (HUVECs; Zhong Sheng, Beijing, China) were thawed and cultured in endothelial cell medium (ECM) supplemented with 10% fetal bovine serum (FBS). The cells were passaged at 80–90% confluence and maintained for subsequent experiments. HUVECs were seeded into 96-well plates at a density of 2000 cells per well in a volume of 100 µL of endothelial cell medium. EK-12 was dissolved in sterile phosphate-buffered saline (PBS) to prepare drug solutions at concentrations of 10–3000 μg/mL, using low (10–250 μg/mL) and high (500–3000 μg/mL) dose ranges to assess dose-dependent effects [23].

HUVECs were seeded into 96-well plates (100 μL/well) and pre-cultured for 24 h in an incubator set at 37 °C and 5% CO_2_ to ensure adhesion. Subsequently, the cells were treated with SMAPs at varying concentrations or with a drug-free medium (control) for 24 h, with six replicates in each group. After treatment, 10 μL of cell counting kit-8 (CCK-8) reagent was added to each well, followed by 2 h of incubation. Absorbance at 450 nm was measured using a microplate reader (Tecan Spark, Männedorf, Switzerland) to calculate cell viability. In this study, the cell proliferation assay and the subsequent cell scratch and tube formation assays were independently repeated more than three times [24].

### 4.5. Cell Scratch Test

For the cell scratch assay performed in 12-well plates, HUVECs were seeded at a density of 2 × 10^5^ cells per well in a total volume of 2 mL of culture medium per well and cultured to 100% confluence. A sterile 200 μL pipette tip was used to create uniform scratches in the monolayer. After washing with PBS to remove detached cells and debris, the experimental wells were treated with SMAPs at designated concentrations (1000, 1500, and 2000 μg/mL), while the controls were treated with a standard medium [24]. Cell migration was monitored at 6, 12, and 24 h post-scratch using an inverted phase-contrast microscope (Leica, Wetzlar, Germany). The cell migration rate was calculated as follows:

$$CM(\%)=\frac{S_{0}-S}{S_{0}}\times 100\%$$

*CM*, cell migration rate; *S*_0_ = initial scratch area; *S* = remaining scratch area at observation time.

### 4.6. Tube Formation Assay

Matrigel (Basement Membrane Matrix, Growth Factor Reduced, BD Biosciences, San Jose, CA, USA, Catalog # 356230) was thawed overnight at 4 °C. It was then diluted with cold serum-free ECM at a 1:1 ratio, and 50 µL was aliquoted into each well of a pre-chilled 24-well plate. The plates were incubated for 30–45 min at 37 °C to allow polymerization. After Matrigel polymerization, HUVECs were trypsinized, resuspended in ECM supplemented with 10% FBS and containing SMAPs (final concentration 1500 µg/mL), and seeded onto the polymerized Matrigel at a density of 1 × 10^4^ cells per well in a final volume of 1 mL. The control group received cells that were resuspended in the same medium without SMAPs. Cells were cultured in an incubator set at 37 °C and 5% CO_2_, and tubular structures were imaged at 6, 12, and 24 h using a microscope. The tube network nodes were quantified using the ImageJ software 1.54f [24].

### 4.7. Pro-Wound-Healing Assay of SMAPs

Healthy adult Kunming male mice (aged 7 weeks and weighing 30–45 g) were obtained from Gema Gene Company (Suzhou, China, with a license SYXK2024-0013). This study was approved by the Ethics Committee of the Medical Center of Soochow University (approval no. 2021-210044), and all procedures complied with the ARRIVE 2.0 guidelines for animal research reporting, including randomization, blinding, and sample size calculations.

The mice were accommodated for a week, anesthetized, and subjected to dorsal hair removal using electric clippers and a depilatory cream. The skin was disinfected using 75% ethanol. Next, 18 full-thickness circular wounds (1 cm in diameter) were established on the dorsum of six mice. The wounds were randomly divided into experimental and control groups, with nine wounds in each group. The experimental group was treated daily with 1500 μg/mL SMAP formulated as a Vaseline-based paste (1500 μg/mL), while the control group received Vaseline alone. The treatments were applied topically to the wound sites. Wound areas were photographed on days 0, 3, 7, 10, and 14 and measured using ImageJ software. The wound healing assay was repeated twice to ensure reproducibility. The healing rate was calculated as follows [7]:

$$HR(\%)=\frac{S_{0}-S}{S_{0}}\times 100\%$$

*HR*, healing rate; *S*_0_, initial wound area; *S*, remaining wound area at the observation time.

### 4.8. Histopathological Examination

After euthanasia, the wound tissues were excised, rinsed with saline, and fixed in paraffin. The sections were stained with hematoxylin and eosin (HE) for histological evaluation. Immunofluorescence staining for CD31 (an endothelial marker) and vascular endothelial growth factor (VEGF) was performed to assess the microvascular density and angiogenesis of the tissue. The primary antibodies used were Anti-CD31 Rabbit pAb and Recombinant Anti-VEGFA antibody (rabbit mAb). The secondary antibody was Cy3-labeled goat anti-rabbit IgG (Servicebio, Wuhan, China).

### 4.9. Detection of VEGF and CD31 Protein Expression Using Western Blotting

Total protein was extracted from frozen wound tissues in each group and quantified using a bicinchonic acid (BCA) kit (Solarbio, Shanghai, China). Equal amounts of protein were separated by SDS-PAGE and transferred onto PVDF membranes. The membranes were incubated sequentially with primary antibodies against CD31 (mouse, 1:1000), VEGF (Servicebio, Wuhan, China, 1:1000), and GAPDH (Abcam, Cambridge, UK, 1:2000) at 4 °C overnight, followed by an HRP-conjugated goat anti-mouse secondary antibody (1:2000) for 2 h at room temperature. After washing with TBST, the protein bands were visualized using enhanced chemiluminescence (ECL, Share-bio, Shanghai, China), and images were obtained [25]. Band intensities were quantified using ImageJ software 1.54f, with GAPDH as the loading control for normalization.

### 4.10. Statistical Analysis

Data were analyzed using SPSS version 27.0. Continuous variables are presented as mean ± standard deviation (SD) for normally distributed data or median (interquartile range, IQR) for non-normally distributed data. Categorical variables are expressed as frequencies (percentages) (n%). Group comparisons were performed using an independent samples *t*-test for continuous data or a chi-squared test for categorical data. Graphs were generated using Origin 2024, with error bars denoting the SD or SEM. Statistical significance is denoted as * *p* < 0.05, ** *p* < 0.01, and *** *p* < 0.001.

## 5. Conclusions

In this study, an active peptide, EK-12, was successfully screened from snail mucus. The amino acid sequence was EAFDDAISELEK, and the molecular weight was 1366.2 Da. Through in vitro and in vivo experiments, we found that EK-12 significantly promoted the proliferation, migration, and tubular structure formation of endothelial cells and accelerated the wound healing process in mice. In addition, pathological examination showed that the expression of CD31 and VEGF in wound tissues treated with EK-12 was significantly upregulated, indicating that it promoted wound healing by regulating angiogenesis-related pathways. Therefore, EK-12, as a natural source of active peptides, has the potential to be developed as a wound healing treatment drug. Future studies will further verify its mechanism of action, including its interaction with VEGFR-2 and its downstream signaling pathways, and explore its anti-inflammatory and other therapeutic mechanisms.

## Data Availability

The original contributions presented in this study are included in the article. Further inquiries can be directed to the corresponding authors.

## Supplementary Materials

The following supporting information can be downloaded at: https://www.mdpi.com/article/10.3390/ijms262110341/s1.

## Abbreviations

The following abbreviations are used in this manuscript:

BCA	Bicinchoninic Acid
CCK-8	Cell Counting Kit-8
CD31	Cluster of Differentiation 31
CM	Cell migration rate
DAPI	4′,6-Diamidino-2-Phenylindole
EC	Endothelial cells
ECL	Enhanced Chemiluminescence
ECM	Endothelial Cell Medium
FBS	Fetal Bovine Serum
GAPDH	Glyceraldehyde-3-Phosphate Dehydrogenase
HPLC	High-Performance Liquid Chromatography
HR	Healing rate
HRP	Horseradish Peroxidase
HUVECs	Human umbilical vein endothelial cells
IHC	Immunohistochemistry
IL-1β	Interleukin-1 Beta
IL-8	Interleukin-8
IL-10	Interleukin-10
KEGG	Kyoto Encyclopedia of Genes and Genomes
MAPK/ERK	Mitogen-Activated Protein Kinase/Extracellular Signal-Regulated Kinase
MS/MS	Tandem mass spectrometry
PI3K/AKT	Phosphoinositide 3-Kinase/Protein Kinase B
PVDF	Polyvinylidene Fluoride
SMAP	Snail mucus active peptides
SDS-PAGE	Sodium Dodecyl Sulfate–Polyacrylamide Gel Electrophoresis
TNF-α	Tumor Necrosis Factor-Alpha
VEGF	Vascular Endothelial Growth Factor
VEGFA	Vascular Endothelial Growth Factor A
